# Proteasome Dependent Actin Remodeling Facilitates Antigen Extraction at the Immune Synapse of B Cells

**DOI:** 10.3389/fimmu.2019.00225

**Published:** 2019-02-19

**Authors:** Jorge Ibañez-Vega, Felipe Del Valle Batalla, Juan José Saez, Andrea Soza, Maria-Isabel Yuseff

**Affiliations:** ^1^Departamento de Biología Celular y Molecular, Facultad de Ciencias Biológicas, Pontificia Universidad Católica de Chile, Santiago, Chile; ^2^Centro de Biología Celular y Biomedicina (CEBICEM), Facultad de Medicina y Facultad de Ciencias, Universidad San Sebastián, Santiago, Chile; ^3^Centro de Envejecimiento y Regeneración (CARE), Facultad de Ciencias Biológicas, Pontificia Universidad Católica de Chile, Santiago, Chile

**Keywords:** B cells, immune synapse, cell polarity lysosomes proteasome, cytoskeleton remodeling, Arp2, MG-132, epoxomicin

## Abstract

Engagement of the B cell receptor (BCR) with surface-tethered antigens leads to the formation of an immune synapse (IS), where cell signaling and antigen uptake are tightly coordinated. Centrosome re-orientation to the immune synapse has emerged as a critical regulatory step to guide the local recruitment and secretion of lysosomes, which can facilitate the extraction of immobilized antigens. This process is coupled to actin remodeling at the centrosome and at the immune synapse, which is crucial to promote cell polarity. How B cells balance both pools of actin cytoskeleton to achieve a polarized phenotype during the formation of an immune synapse is not fully understood. Here, we reveal that B cells rely on proteasome activity to achieve this task. The proteasome is a multi-catalytic protease that degrades cytosolic and nuclear proteins and its dysfunction is associated with diseases, such as cancer and autoimmunity. Our results show that resting B cells contain an active proteasome pool at the centrosome, which is required for efficient actin clearance at this level. As a result of proteasome inhibition, activated B cells do not deplete actin at the centrosome and are unable to separate the centrosome from the nucleus and thus display impaired polarity. Consequently, lysosome recruitment to the immune synapse, antigen extraction and presentation are severely compromised in B cells with diminished proteasome activity. Additionally, we found that proteasome inhibition leads to impaired actin remodeling at the immune synapse, where B cells display defective spreading responses and distribution of key signaling molecules at the synaptic membrane. Overall, our results reveal a new role for the proteasome in regulating the immune synapse of B cells, where the intracellular compartmentalization of proteasome activity controls cytoskeleton remodeling between the centrosome and synapse, with functional repercussions in antigen extraction and presentation.

## Introduction

B Lymphocytes mediate humoral responses by the recognition of antigens tethered at the surface of presenting cells such as follicular dendritic cells or macrophages, forming a domain known as an Immune Synapse (IS) (1–3). Immune synapse formation is initiated upon recognition of surface-tethered antigens by the B cell receptor (BCR), triggering a rapid actin–dependent membrane spreading response (4) where antigen-BCR complexes are gathered into micro-clusters that contain signaling molecules, such as tyrosine kinases, Lyn, and Syk (1). The spreading reaction exerted by B cells is tightly coupled to their signaling capacity, as cells that recruit fewer signaling molecules to micro-clusters show deficient spreading responses to membrane-bound antigens (4). This is followed by a contraction phase where BCR-Ag complexes are concentrated into a central cluster by the concerted action of the microtubule-based motor protein, dynein, and actin rearrangements (4). Extracellular cues, such as the physical properties of the environment can determine whether antigens are mechanically or proteolytically captured from different surfaces (5). The extraction of antigens immobilized on stiffer substrates can be facilitated by proteases, which originate from the local secretion of MHC-II^+^ lysosomes at the synaptic membrane. This process is controlled by the cooperative action of polarity complex proteins, such as Cdc42, Par3, and aPKC, which promote the recruitment of the centrosome and associated lysosomes toward the immune synapse (6–8). The peptides generated from uptaken antigens are further mounted onto MHC-II molecules and presented to T cells in order to trigger B-T cooperation, thereby promoting the maturation and differentiation of B cells to memory B cells or plasma cells (9, 10).

Alterations during the activation of B cells have been associated to autoimmune disorders, such as Systemic Lupus Erythematosus (SLE), Rheumatoid Arthritis (RA), and Sjögren's Syndrome (10–12). Deregulation of BCR signaling is associated to a breach in B cell tolerance, leading to a defective selection of naïve self-reactive B cells and/or the formation of autoantibody-producing plasma cells (13). Drugs that target the development of plasma cells, such as Bortezomib, are frequently used as a treatment against SLE and have been shown to ameliorate clinical manifestations of refractory SLE (14). Bortezomib specifically binds to the 26S proteasome and inhibits its chymotrypsin-like protease activity. Plasma cells treated with bortezomib accumulate misfolded proteins, which blocks the antiapoptotic pathway of NF-κB, leading to cell death (14). However, the molecular mechanisms underlying the effects of proteasome inhibitors in the activation, differentiation and survival of immune cells remain poorly studied.

The proteasome is a complex molecular system that degrades cytosolic proteins upon their conjugation to ubiquitin (15). Proteolysis via the ubiquitin-proteasome-system (UPS) is an efficient method to control levels of specific proteins, involved in cell signaling, cell division, polarity, and cytoskeleton remodeling, in time and space (16–19). The 26S proteasome is composed of two major subunits: the 19S Regulatory particle (RP) and the 20S proteasome or catalytic core (20), which together give rise to a 26S structure. The core particle (20S proteasome) requires the regulatory particle (19S) to degrade ubiquitinated protein substrates (20). There are several reports that indicate that the UPS can regulate actin dynamics and control cell polarity (21–23). For instance in neurons, the proteasome is actively depleted from the growth cones in order to increase the accumulation of proteins that trigger cytoskeleton polymerization and thereby promote their extension (24). Moreover, inhibition of the proteasome triggers the formation of multiple axons, and thus neurons lose their polarized phenotype (25). In T lymphocytes, asymmetric segregation of the proteasome into daughter cells, mediated by the polarity protein aPKC, is required to promote their differentiation (16). During primary cilia formation, a related molecular model of an immune synapse (26), proteasome activity regulates the composition of centrosome associated proteins, thereby controlling initial stages of axoneme extension (27). Thus, the UPS is widely used to shape polarized membrane dynamics, however its role in B cells has not been addressed so far. In this study, we explored whether the polarized distribution of the proteasome and its activity regulates the establishment of a functional immune synapse. We show here that resting B cells accumulate active proteasome at their centrosome, which is recruited to the immune synapse upon activation. Pharmacological inhibition of the proteasome, using MG-132 or Epoxomicin, leads to an accumulation of actin at the centrosome, impairing its detachment from the nucleus and subsequent re-positioning to the IS together with lysosomes. Consequently, under these conditions, antigen extraction, and presentation are significantly impaired. Inhibition of proteasome activity is also associated with a diminished recruitment of actin and Arp2/3 at the synaptic membrane, leading to a defective spreading response and distribution of signaling proteins in B cells. Overall our results unveil how local proteostasis regulated by the proteasome controls the formation of an immune synapse and B cell responses to immobilized antigens.

## Materials and Methods

### Mice, Cell Lines, Culture, and Treatments

Resting mature spleen IgM^+^IgD^+^ B cells were purified from 8 to 12 weeks old C57BL/6 mice by negative magnetic selection as previously described (28). The mouse lymphoma cell line IIA1.6 is a FcγR-defective B cell line with the phenotype of quiescent mature B-cells (29) and the LMR7.5 Lack T-cell hybridoma which recognizes I-Ad-LACK_156–173_ complexes, were cultured as previously described (28) in CLICK medium (RPMI 1640, 10% fetal bovine serum, 1% penicillin–streptomycin, 0.1% β-mercaptoethanol, and 2% sodium pyruvate). For proteasome inhibition, 5 × 10^6^ B cells/mL were incubated with 5 μM MG-132 or 10 μM Epoxomicin for 1 h at 37°C before functional analysis.

### Antibodies and Reagents

We used Rat anti-mouse LAMP1 (BD Bioscience, San Jose, CA), Rabbit anti-mouse α-Tubulin (Abcam), Rabbit anti-mouse γ-Tubulin (Abcam), Rabbit anti-mouse S4/19S RP (Abcam), Rabbit anti-mouse αβ/20S proteasome (Abcam), Rabbit anti-mouse α4/20S proteasome (Abcam), Mouse anti-mouse Ubiquitin P4D1 (Santa Cruz), Rabbit anti-mouse pSyk (Y525/526) (cellsignalling), Rabbit anti-mouse Syk (Abcam), anti-mouse β-actin (Abcam), Rat anti-mouse CD45R (BD Bioscience), Goat anti-mouse IgGFab^2^ (Jackson ImmunoResearch), Goat anti-mouse IgM Fab^2^ (Jackson ImmunoResearch). For secondary antibodies: Donkey anti-rabbit IgG-Alexa488 (LifeTech), Goat anti-rabbit IgG-Alexa546 (ThermoScientific), Donkey anti-rat IgG-Alexa546 (ThermoScientific), Donkey anti-rat-Alexa647 (ThermoScientific), Phalloidin-Alexa647 (ThermoScientific), DAPI (Abcam). Ovalbumin was purchased from Sigma-Aldrich, MG-132 and Epoxomicin were purchased from Merk (Millipore).

### Cell Transfection

LifeAct-mCherry plasmids were kindly provided by Ana Maria Lennon. Nucleofector R T16 (Lonza, Gaithersburg, MD) was used to electroporate 5 × 10^6^ IIA1.6 B Lymphoma cells with 2 μg of plasmid DNA. After transfection, cells were cultured for 16 h before functional analysis.

### Preparation of Ag-Coated Beads and Ag-Coated Cover-Slides

Antigen coated beads were prepared as previously described (7). Briefly, ~2 × 10^7^ 3-μm latex NH_2_-beads (Polyscience, Eppelheim, Germany) were activated with 8% glutaraldehyde for 4 h at room temperature. Beads were washed with phosphate-buffered saline (PBS) and incubated overnight at 4°C with different ligands using 100 μg/mL of either F(ab′)2 goat anti-mouse immunoglobulin G (IgG), referred to as BCR-Ligand^+^ or F(ab′)2 goat anti-mouse IgM, referred to as BCR-Ligand^−^ (MP Biomedical, Santa Ana, CA). For antigen extraction assays beads were coated with BCR-Ligand^+^ or BCR-ligands^−^ plus OVA 100 μg/mL. For antigen presentation assays, beads were coated with BCR^+^ or BCR^−^ ligands plus 100 μg/mL Lack protein. Antigen-cover-slides used to analyze the synaptic interface were coated with BCR-Ligand^+^ and 0.5 μg/mL B220 (anti-Mouse CD45R) (BD Bioscience) overnight at 4°C in PBS.

### Activation of B Cells With Antigens-Coated Beads or Cover-Slides

Cells were plated on poly-L-Lysine–coated glass coverslips and activated with Ag-coated beads (1:1 ratio) for different time points in a cell culture incubator (37°C/5% CO_2_) and then fixed in 4% paraformaldehyde (PFA) for 10 min at room temperature as previously described (7). Fixed cells were incubated with antibodies in PBS-0.2% BSA-0.05% Saponin. To measure cell spreading, the B cell line or primary B cells were plated onto B220/anti-IgG or anti-IgM coated glass coverslips, respectively, for different time points at 37°C in a cell culture incubator as previously described (6).

### Ag Presentation Assays

Ag presentation assays were performed as previously described (7). Briefly, IIA 1.6 (I-A^d^) B cells were incubated with either Lack-BCR-Ligand^+^ or BCR-Ligand^−^ coated beads or different concentrations of Lack peptide (Lack_156−173_) for 1 h. Then Cells were washed with PBS, fixed in ice-cold PBS/0.01% glutaraldehyde for 1 min and quenched with PBS/100 mM glycine. B cells were then incubated with Lack-specific LMR 7.5 T Cells in a 1:1 ratio for 4 h. Supernatants were collected and interleukin-2 cytokine production was measured using BD optiEA Mouse IL-2 ELISA set following the manufacturer's instructions (BD Biosciences).

### Ag Extraction Assays

For antigen extraction assays, B cells incubated in a 1:1 ratio with BCR ligand^+^-OVA-coated beads were plated on poly-Lys cover-slides at 37°C, fixed and stained for OVA. The amount of OVA remaining on the beads was calculated by establishing a fixed area around beads in contact with cells and measuring fluorescence on three-dimensional (3D) projections obtained from the sum of each plane (Details in Image Analysis section). The percentage of antigen extracted was estimated by the percentage of fluorescence intensity lost by the beads after 1 h.

### Purification of Synaptic Membranes

Synaptic membranes were isolated from B cells using a previously described protocol (30). Briefly, magnetic NH_2_-beads (Dynabead™ M-270 Amine, Invitrogen) were coated with BCR-Ligands+ and incubated with 1 × 10^7^ B cells at a 1:1 ratio in CLICK-2%FBS at 37°C/5% CO_2_ for different time points. Activation was stopped by adding ice-cold PBS and centrifuging samples at 600 g for 5 min at 4°C. Cells were resuspended in cold-PBS, collecting 10% for input controls. All samples were precipitated by magnetic field, removing the supernatant and replacing it with freeze-thaw buffer (600 mM KCl, 20% glycerol, 20 mM Tris-HCl pH 7.4) supplemented with 5 mM NaF, 1 mM Na_3_VO_4_, and complete Mini-protease inhibitor cocktail (Roche, Basel, Switzerland). The samples were frozen and thawed 7 times at −80 and 42°C, respectively. Two microliter benzonase Nuclease (Sigma-Aldrich) was then added and incubated for 30 min. The samples were magnetically precipitated for 5 min and washed 5 times with cold-freeze-thaw buffer, and finally resuspended in loading buffer for SDS-PAGE.

### Centrosome Isolation

Centrosome from B cells were isolated as previously described (31) with slight modifications. Briefly, activated B cells with BCR-Ligand+ coated beads in CLICK-2% FBS (ratio 1:1) were incubated for 60 min at 37°C/5%CO_2_, adding 2 μM cytochalasin D (Merck Millipore) and 0.2 μM Nocodazole (Merck Millipore). Cells were washed in TBS (10 mM Tris-HCl 15 mM NaCl pH 7.5), then in 0.1X TBS supplemented with 8% sucrose and lysed in lysis buffer (1 mM HEPES. 0.5% NP-40, 0.5 mM MgCl_2_, 0.1% β-mercaptoethanol pH 7.2) supplemented with protease inhibitors for 15 min. Centrosomes were isolated from post-nuclear-supernatants by consecutives centrifugations at (1) 10,000 g for 30 min at 4°C on top of a 60% w/v sucrose cushion in gradient buffer (10 mM PIPES, 0.1% Triton X-100, 0.1% β-mercaptoethanol pH 7.2) and (2) 40,000 g for 60 min at 4°C on top of a discontinuous sucrose gradient (40–50–70% w/w). Finally, 12 fractions were recovered from the top to the bottom of the tube, and centrosome-containing fractions were detected by immunoblot.

### Measurement of Proteasome Activity

Protein extracts obtained from B cells were quantified and loaded onto black MaxiSorp 96 well plate (Nunc, Denmark) with Proteasome substrate III fluorogenic (Calbiochem, Merck Millipore) diluted in Assay Buffer (50 mM Tris-HCl pH: 7.2, 0.05 mM EDTA, 1 mM DTT). The plate was incubated for 1 h at 37°C and then fluorescence was measured at 360/420 nm. All measurements were performed in triplicate.

### Epifluorescence Microscopy

All Z-stack images were obtained with 0.5 microns between slices. Images were acquired in an epifluorescence microscope (Nikon Ti2Eclipse) with a X60/1.25NA and X100/1.3NA oil immersion objectives for bead and spreading assays, respectively.

### Confocal Microscopy

Images were acquired in a Nikon Ti2Eclipse inverted microscope with 60X/1.45NA oil immersion for bead and spreading assays, with Z-stack of 0.5 microns.

### TIRFM

Total internal reflection fluorescence microscopy (TIRFM) images were acquired in Nikon Ti2Eclipse inverted microscope with a 100x/1.50 NA oil immersion lens and a iXON Ultra EMCCD camera at 37°C. B-cells expressing LifeAct-mCherry were plated on Ag-coated glass chambers (Nunc™ Lab-Tek™ II). Images were acquired for 30 min at 15 s per frame for spreading.

### Image Analysis

Image processing and analysis was performed with FIJI (ImageJ) software (32). The centrosome was labeled with α-Tubulin and determined by the brightest point where microtubules converged. Single-cell images shown in the figures were cropped from larger field. Images brightness and contrast was manually adjusted. Centrosome polarity index was determined as previously described (7). Briefly, we manually selected the location of centrosome (Cent) and delimited the cell border and bead to obtain the center of mass of both, CMC (Cell mass center) and BMC (Bead mass center), respectively. The position of the centrosome was projected (CentProj) on the vector defined by CMC-BMC axis. The centrosome polarity index was calculated by dividing the distance between the CMC and CentProj and the distance between CMC-BMC. The index ranges from −1 (anti-polarized) and +1 (fully polarized).

Proteasome recruitment to the IS in bead assays was quantified by dividing the fluorescence at the bead by the fluorescence of the whole cell and then multiplying it by 100%. For spreading assays, we manually delimited the border of the cell using phalloidin label as template (CellTemp), then an ellipse was automatically determined (CenterTemp) at the center of CellTemp, which had a third of the CellTemp area. The periphery area (PeripheryTemp) of the IS was calculated by subtracting the CenterTemp to the CellTemp area. Then, the recruitment to the periphery was calculated by dividing the fluorescence normalized by its area from PeripheryTemp and CellTemp, subtracting 1. Therefore, positive values mean that the fluorescence is enriched at the periphery and negative values, the opposite.

For actin quantification at the centrosome we used a 1 μm radium circle considering its center as the centrosome. We quantified the fluorescence at the centrosome (FCent) and its Area (ACent). The corresponding ratio gives fluorescence density index (DCent = FCent/ACent). This value is divided by the density of fluorescence of the entire cell (DCell). Values above 1 indicate that there is an accumulation of the label at the centrosome compared to the whole cell, otherwise, values below 1 indicate that there is a depletion at the centrosome compared to the whole cell. To measure the distance between centrosome and nucleus, we considered the location of the centrosome (Cent) and the mass center of the nucleus (MCN), measuring the distance between both points in μm.

### Statistical Analysis

Statistical analysis was performed with Prism (GraphPad Software). The *p*-values were computed using different tests as indicated in figure legends; ^*^0.01 < *p* < 0.05, ^**^0.001 < *p* < 0.01; ^***^p < 0.001; ns, no significant.

## Results

### Proteasome Activity Is Required for Efficient Extraction and Presentation of Immobilized Antigens by B Cells

**Figure 1:**
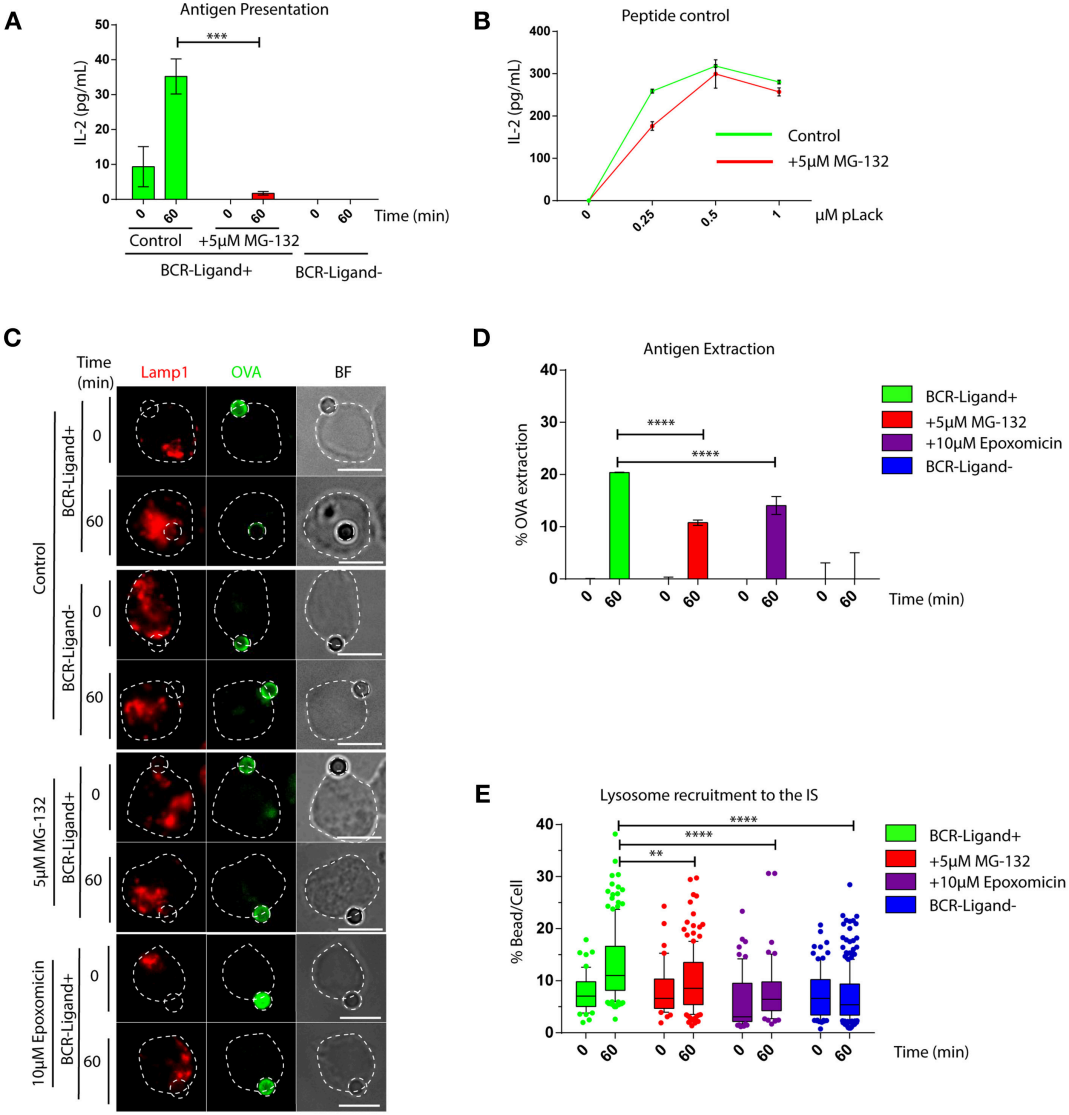
The capacity of B cells to extract and present extracellular antigens relies on proteasome activity. **(A)** Antigen presentation assay for control and MG-132 pre-treated cells. Levels of IL-2 secretion by T cells were quantified by ELISA. ^***^p < 0.001. *N* = 3. **(B)** Representative graph of peptide controls for cells used in antigen presentation assays. **(C)** Representative images of control, MG-132 and Epoxomicin pre-treated cells incubated with beads coated with anti-IgG+OVA (BCR-Ligand+) or anti-IgM+OVA (BCR-Ligand–) in resting (0 min) and activated (60 min) conditions. Fixed cell-bead conjugates were stained for OVA (green) and LAMP-1 (red). Scale bar = 10 μm. **(D)** Antigen extraction was measured as the amount of OVA extracted from the bead (see Materials and Methods). ^****^p < 0.001. *N* = 4 (>100 cells). **(E)** Lysosome recruitment to the bead during B cell activation in control, MG-132 and Epoxomicin pre-treated cells. ^****^p < 0.001, ^**^0.001 < *p* < 0.01. *N* = 4 (>100 cells). 2-way ANOVA with Sidak's *post-test* was performed for all statistical analysis. Mean with SEM bars are shown.

We first investigated whether an acute inhibition of proteasome activity had an impact in the capacity of B cells to extract and present immobilized antigens. For this purpose, we pretreated B cells with 5 μM MG-132 for 1 h, which reduces approximately 80% of proteasome activity and leads to an increase in ubiquitylated proteins (Figures S1A,B) without affecting cell viability (Figure S1C). Antigen presentation assays using B cells pre-treated or not with MG-132 revealed that there was a significant reduction in the capacity of B cells to present immobilized antigens to T cells when the proteasome was inhibited (Figure 1A), whereas peptide presentation showed no major differences between both conditions (Figure 1B). These results indicate that inhibition of proteasome activity in B cells does not affect cell surface levels of MHC-II molecules and does not influence B-T cell interactions *per se*, but could affect critical steps upstream of antigen presentation, such as antigen extraction and processing. To this end, antigen extraction was evaluated by measuring the fluorescence signal of ovalbumin (OVA) remaining on activating (BCR-ligand^+^) or non-activating (BCR-ligand^−^) beads after their interaction with B cells pre-treated or not with MG-132. As anticipated, B cells pretreated with MG-132 extracted less antigen compared to their control counterparts (Figures 1C,D), indicating that antigen extraction is partially proteasome-dependent. To discard that our observations were due to off target effects elicited by MG-132, we evaluated the antigen extraction capacity of B cells pretreated with another proteasome inhibitor, Epoxomicin. Treatment of B cells with 10 μM of Epoxomicin lead to an accumulation of ubiquitylated proteins without affecting cell viability (Figures S1D,E). Under these conditions, B cells also displayed defects in the extraction of OVA from activating latex beads (Figures 1C,D), thus confirming that proteasome inhibition impairs the ability of B cells to efficiently extract immobilized extracellular antigens. Given that antigen extraction from rigid surfaces, such as latex beads, relies on lysosome secretion at the synaptic interface (3, 7), we next evaluated whether lysosome recruitment to the immune synapse was also impaired when proteasome activity was inhibited. Consistent with our findings showing defects in antigen extraction, we observed that lysosomes were not recruited to the immune synapse when proteasome activity was inhibited using MG-132 or Epoxomicin (Figure 1E) and instead remained confined to the cell center (Figure 1C). Similar effects were observed in primary B cells pretreated with proteasome inhibitors, which failed to efficiently extract OVA from activating from latex beads (Figures S1F,G), showing that proteasome activity also regulates antigen extraction in naïve B cells.

Together our data show that proteasome activity is required for efficient lysosome recruitment to the IS and thereby regulates the extraction and presentation of extracellular antigens by B cells.

### Clearance of Centrosome-Associated F-Actin and Lymphocyte Polarity Depend on Proteasome Activity

**Figure 2:**
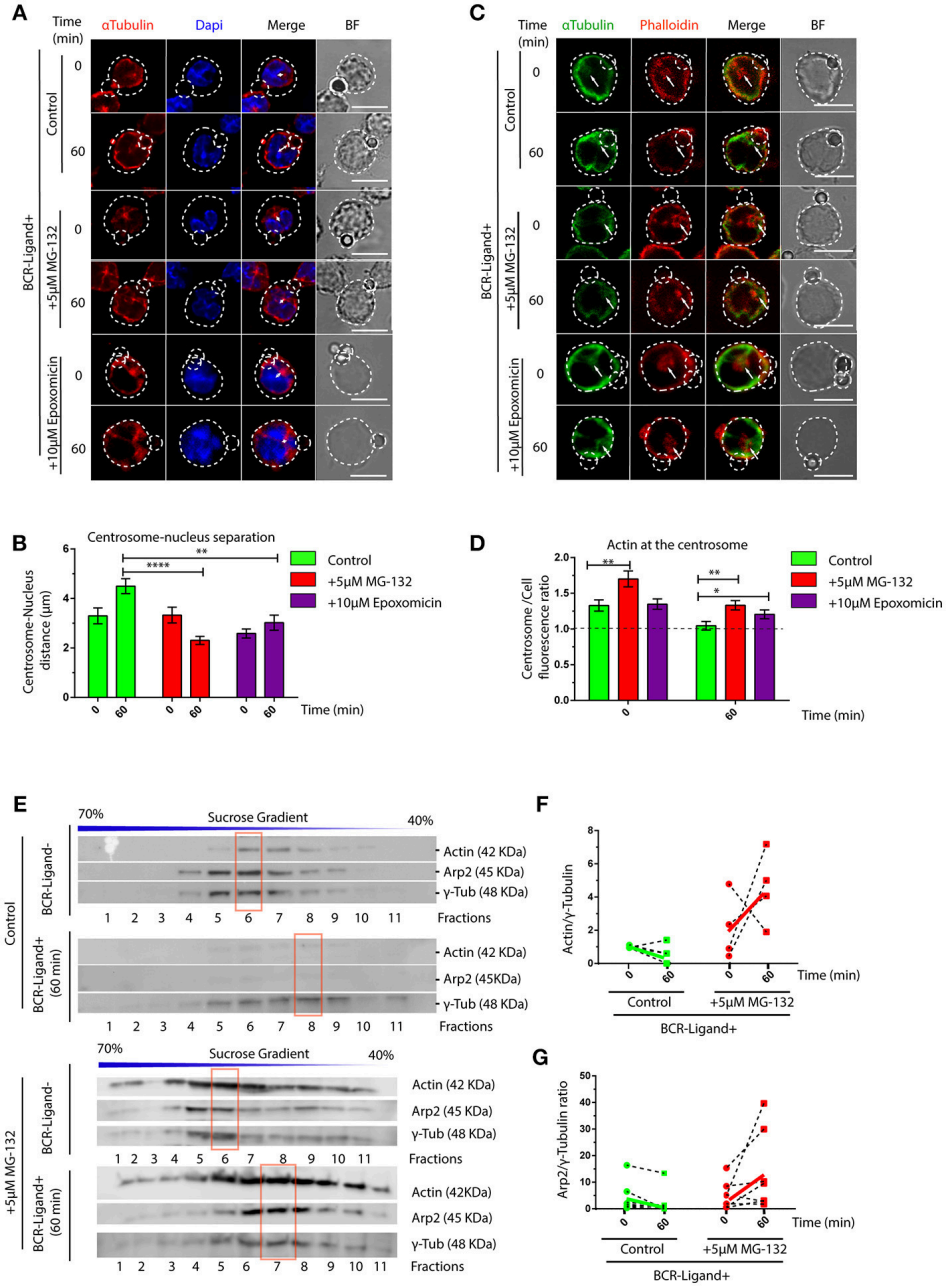
Proteasome activity controls centrosome detachment from nucleus by actin clearance. **(A)** Representative images of control, MG-132 and Epoxomicin pre-treated B cells incubated with BCR-Ligand+ coated beads at 0 and 60 min. Cells were stained for α-Tubulin (red), and DAPI (blue). Distance between the centrosome and nucleus mass center is illustrated (white double-arrow). Scale bar = 10 μm. **(B)** Centrosome-Nucleus distance measurements for images in A. ^****^p < 0.001, ^*^p < 0.05. *N* = 4 (>40 cells). **(C)** Representative images of control, MG-132 and Epoxomicin pre-treated B cell during activation. α-Tubulin (green) and Phalloidin (red). White arrows indicate centrosome localization. Scale bar = 10 μm. **(D)** Actin mass at the centrosome quantified by immunofluorescence (see Materials and Methods). ^**^0.001 < *p* < 0.01. *N* > 5 (>100 cells). **(E)** Immunoblot of centrosome fractions obtained from control and MG-132 pre-treated B cells at 0 and 60 min post activation. Actin, Arp2, and γ-tubulin were detected in each fraction. Red rectangles indicate the fraction with highest γ-tubulin levels. **(F,G)** Rate of Actin and Arp2 change at the centrosome fraction after 60 min of activation. (*N* = 5), respectively. 2-way ANOVA with Sidak's *post-test* was performed for all statistical analysis. Mean with SEM bars are shown.

We next searched for the cellular basis underlying defective lysosome recruitment and antigen extraction in B cells treated with proteasome inhibitors and focused on mechanisms that regulate B cell polarity. Given that the transport of lysosomes to the IS relies on the polarization of the centrosome, we measured re-positioning of this organelle to the synaptic membrane in activated B cells pre-treated or not with MG-132 or Epoxomicin. Indeed, we observed that inhibition of proteasome activity impaired the polarization of the centrosome to the immune synapse of activated B cells (Figure S2), where it remained more confined to the cell center and close to the nucleus (Figures 2A,B). A recent study showed that in B cells, clearance of F-actin at the centrosome allows its detachment from the nucleus and polarization to the immune synapse (31). We therefore hypothesized that actin clearance at the centrosome might be impaired in B cells treated with proteasome inhibitors. To test this hypothesis, we performed immunofluorescence staining of microtubules and actin in resting and activated B cells pre-treated or not with MG-132 or Epoxomicin and measured the amount of actin around the centrosome (Figures 2C,D). Indeed, we observed significantly higher levels of actin at the centrosome in both resting and activated B cells when the proteasome was inhibited compared to control counterparts. Upon activation, actin was partially depleted from the centrosome of proteasome-inhibited B cells, however overall levels were still comparable to those of resting control B cells (Figure 2D). This suggests that depletion of F-actin at the centrosome, triggered upon B cell activation, is not sufficient when proteasome activity is inhibited and thus B cells cannot uncouple their centrosome from the nucleus. We next decided to confirm the effect of proteasome inhibition on centrosome-associated F-actin using a biochemical approach. For this purpose, centrosome-enriched fractions were purified from resting and activated B cells previously treated or not with MG-132 and actin levels quantified by immunoblot. In agreement with our image analysis, actin was depleted from centrosome rich fractions upon BCR stimulation (Figure 2E, upper panel and Figure 2F). However, this was not observed in activated B cells previously treated with MG-132, where actin levels even increased in centrosome fractions compared to the resting state (Figure 2E, lower panel and Figure 2F). Similarly to actin, a subunit from the Arp2/3 complex, Arp2, involved in actin nucleation at the centrosome also accumulated in centrosome fractions when proteasome activity was inhibited and did not decrease upon BCR engagement (Figure 2G). Thus, our data show that proteasome activity regulates the levels of F-actin at the centrosome of B cells.

### Proteasome Activity Controls Actin Dynamics and Signaling at the Immune Synapse

**Figure 3:**
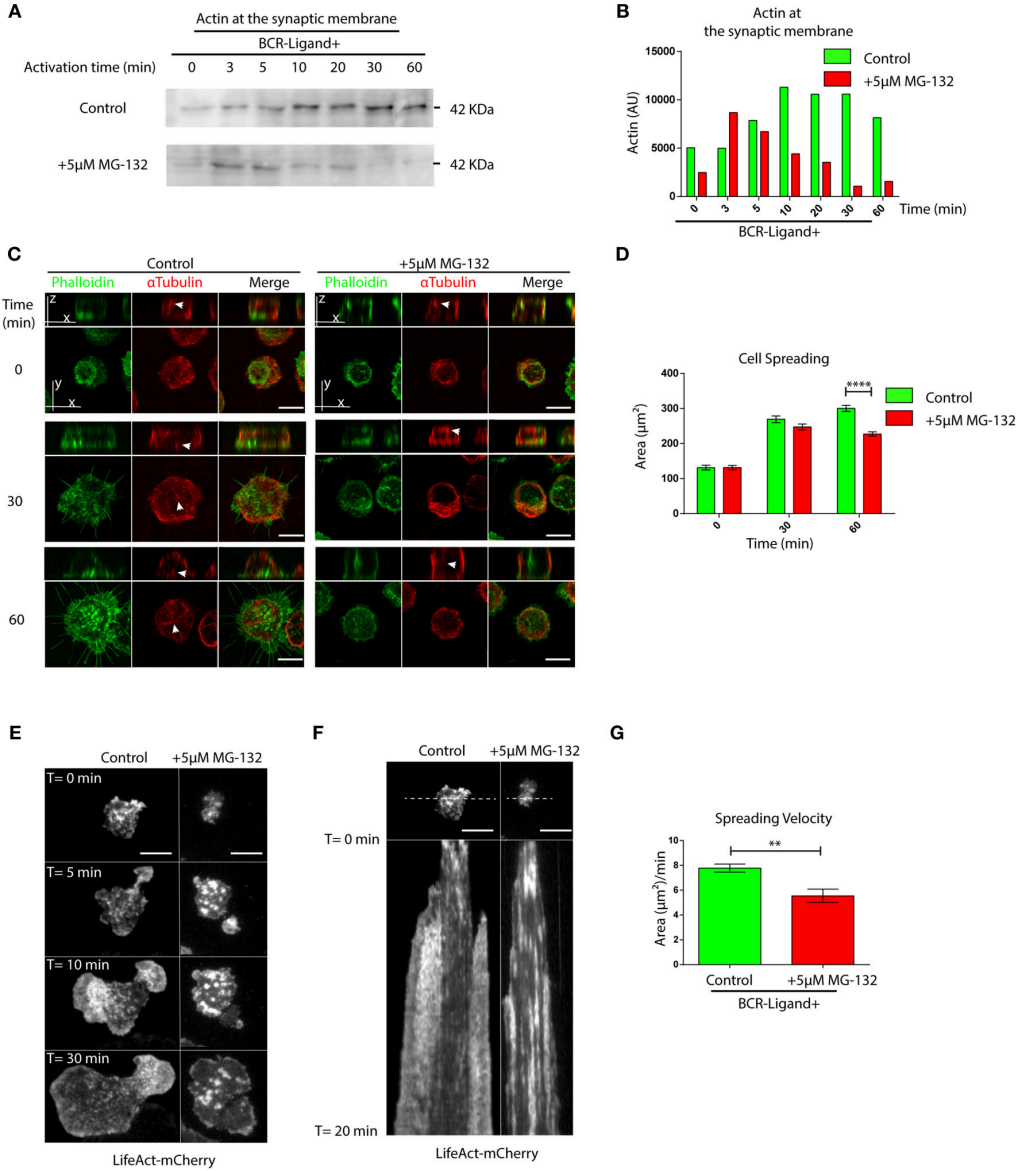
Proteasome activity controls actin remodeling at the immune synapse. **(A)** Immunoblot showing actin levels in synaptic membranes obtained from control and MG-132 treated B cells after different time points of activation. **(B)** Quantification of actin at the synaptic membrane. **(C)** Representative images of control and MG-132 treated B cells activated on antigen-coated cover-slides for different time points. F-actin (green) and α-Tubulin (red). White arrows indicate centrosome localization. Scale bar = 10 μm. **(D)** Quantification of the spreading area of B cells pre-treated or not with MG-132 and activated for different time points ^****^p < 0.001. *N* = 5 (>200 cells). 2-way ANOVA with Sidak's *post-test* was performed. **(E)** Time-lapse images of Lifeact-mCherry expressing B cells pretreated or not (control) with MG-132 activated on antigen-coated coverslides. Scale bar = 10 μm. **(F)** Kymograph of time vs. distance obtained from a 20-min movie generated at 1 frame per 10 s showing the first 20 min of actin distribution (white) tracked by LifeAct-mCherry. Scale bar = 10 μm. **(G)** Velocity of spreading in control and MG-132 treated B cells expressing LifeAct-mCherry (μm^2^/min). ^**^0.001 < *p* < 0.01. Student's *t*-test. *N* = 6. Mean with SEM bars are shown.

Having shown that proteasome inhibition leads to an accumulation of actin and Arp2 at the centrosome of B cells, we next evaluated whether this has consequences in their recruitment to the immune synapse. Indeed, when we isolated synaptic membranes from activated B cells, we observed a reduced recruitment of actin to the IS when proteasome activity was inhibited (Figures 3A,B). Accordingly, image analysis revealed that Arp2 and actin failed to reach the immune synapse and remained confined to the center of the cell when proteasome activity was inhibited (Figures S3A,B). We therefore decided to evaluate overall actin remodeling at the immune synapse by imaging the synaptic interface of B cells activated on antigen-coated cover-slides. Indeed, B cells treated with MG-132, showed defects in the accumulation of actin at the periphery and synapse center as well as less filopodia-like structures compared to control conditions (Figure 3C). Consistent with this observation, MG-132 treated B cells also displayed defective spreading capacity (Figures 3C,D), which was also observed in primary B cells pre-treated with MG-132 (Figures S3C,D). To further characterize whether actin dynamics was affected by proteasome inhibition, we seeded B cells expressing LifeAct-mCherry, pre-treated or not with MG-132 and analyzed the synaptic membrane area by TIRFM (Videos S1, S2). In agreement with results obtained with fixed cells, MG-132 treated B cells reduced their spreading velocity (Figures 3E–G), thereby confirming that proteasome activity controls actin dynamics at the immune synapse during B cell activation.

**Figure 4:**
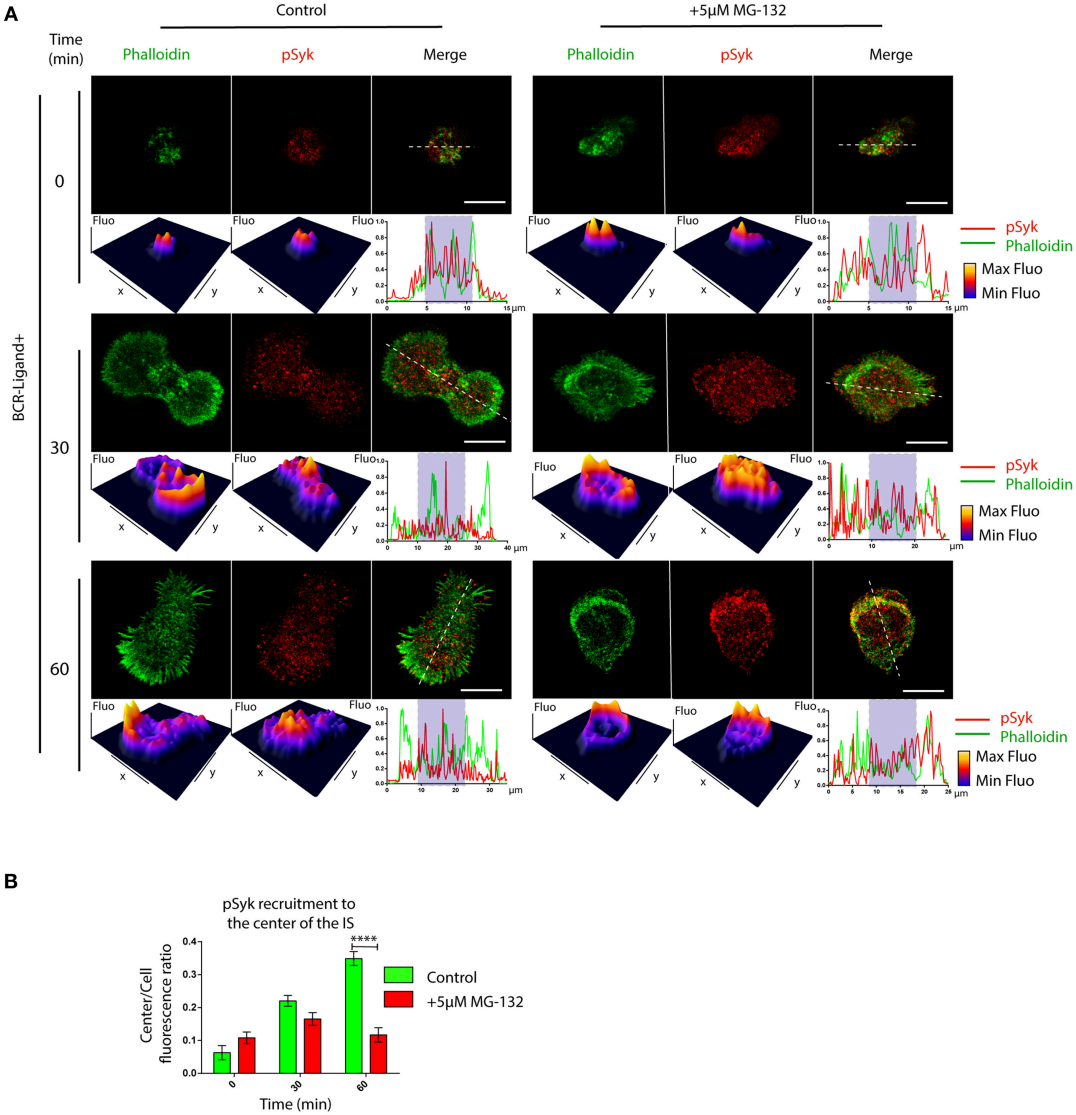
Inhibition of proteasome activity decreases the recruitment of phospho-Syk at the center of the immune synapse. **(A)** Representative confocal images of control and MG-132 treated B cells activated on antigen coated coverslides for different time points. Fluorescence intensity distribution of Phalloidin (green) and pSyk (red) was 3D plotted and quantified (side graphs) across the cell (dashed white lines). Rectangle in graphs, shown below images represent the central synaptic area. Scale bar = 10 μm. **(B)** Quantification of pSyk at the center of the IS for both conditions and different time points. ^****^p < 0.001 (>70 cells). 2-way ANOVA with Sidak's *post-test*. Mean with SEM bars are shown.

BCR signaling is coupled to actin cytoskeleton remodeling at the IS (33). This prompted us to determine whether B cell signaling was also affected when the proteasome is inhibited. We first analyzed the levels and distribution of phosphorylated Syk (pSyk), a direct BCR downstream signaling molecule, at the synaptic membrane by confocal microscopy in B cells activated by antigens immobilized on cover-slides. In control B cells, we found that pSyk accumulates inside the boundaries of the peripheral actin ring at the immune synapse (Figure 4A). In contrast, the distribution of pSyk in MG-132 treated B cells was dispersed throughout the synaptic membrane and was localized beyond the boundaries of the actin ring (Figures 4A,B). Notably, synaptic membranes isolated from activated B cells at different time points revealed that higher levels of Syk were associated to the immune synapse when the proteasome was inhibited (Figures S4A,B). This effect most likely results from impaired degradation of activated Syk mediated by the ubiquitin-proteasome system (34). However, the levels of pSyk remained similar to control conditions, producing a lower ratio pSyk/Syk in MG-132 pre-treated B cells (Figures S4A,C), revealing that higher levels of Syk are not associated to its stable phosphorylation. Overall, these results suggest that proteasome activity does not significantly affect BCR signaling, but rather the localization of signaling components at the immune synapse.

### The Proteasome Is Actively Recruited to the Immune Synapse

**Figure 5:**
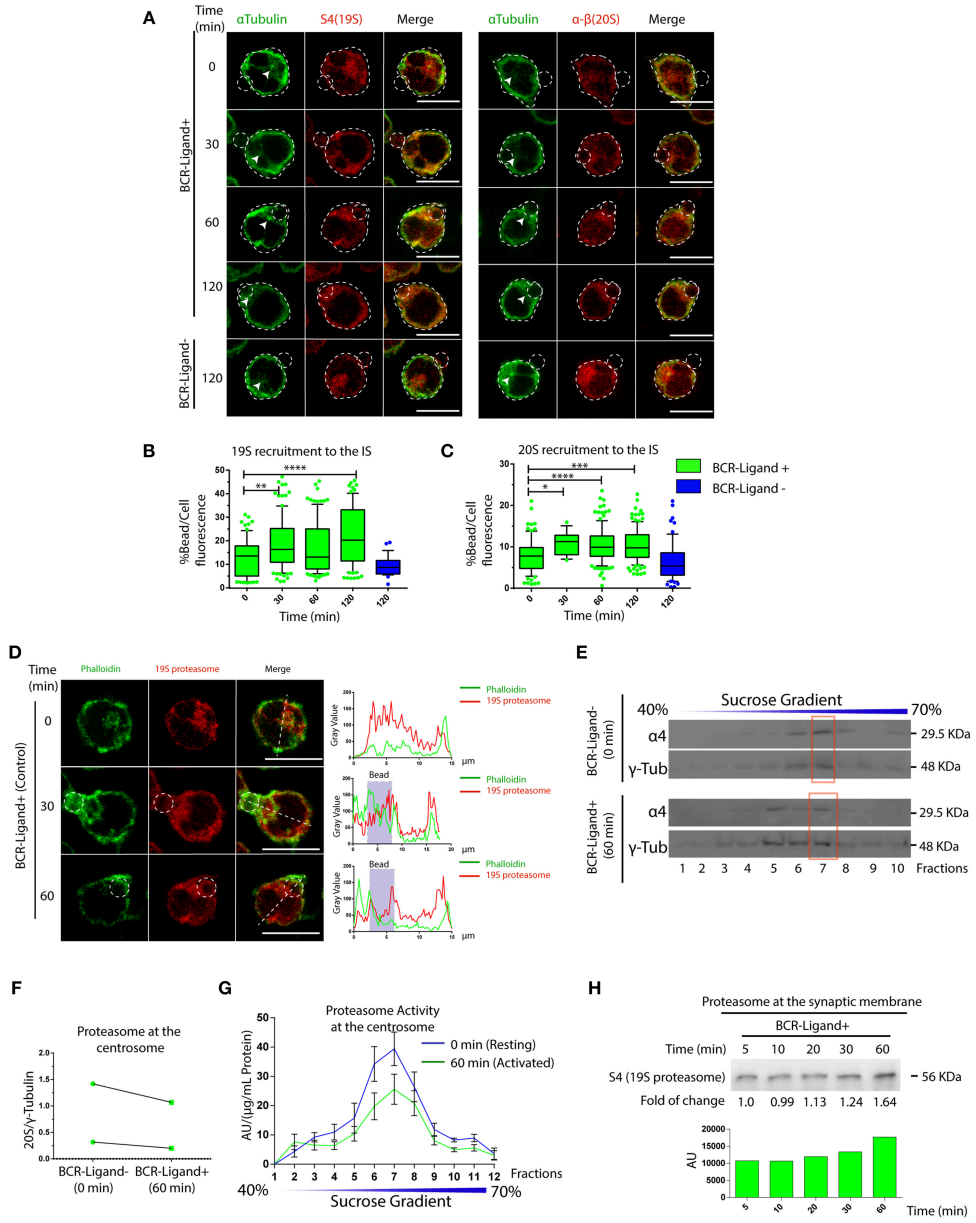
Dynamics of proteasome recruitment to the Immune synapse. **(A)** Representative images of B cells activated with BCR-Ligand+ or BCR-Ligand– coated beads for different time points. α-Tubulin (green), S4 (19S RP subunit [red]) and α-β (20S proteasome subunits [red]-right panel). Scale bar = 10 μm. **(B,C)**. Quantification of 19S RP and 20S proteasome recruitment to the bead (antigen contact site) during B cell activation, respectively. ^*^0.01 < *p* < 0.05, ^**^0.001 < *p* < 0.01, ^***^p < 0.001, ^****^p < 0.001. *N* = 5 (>200 cells). **(D)** Representative confocal images of B cells activated with antigen-coated beads for different time points. Actin (green) and 19S RP (red) were stained. Graphs on the right show the distribution of actin (green line) and proteasome (red line) across the cell (dashed white line). Scale bar = 10 μm. **(E)** Immunoblot of centrosome fractions obtained from resting (0 min) and activated (60 min) B cells. 20S proteasome and γ-tubulin proteins were detected. Red rectangles indicate the centrosome fraction with highest levels of γ-tubulin. **(F)** Quantification of 20S proteasome (α4) in centrosome-rich fractions (red rectangle from blot in **(E)**, expressed as the ratio between α4 and γ-tubulin levels (*N* = 2). **(G)** Quantification of the proteasome protease-activity at each fraction obtained from resting and activated B cells. (*N* = 5). **(H)** Immunoblot of B cell synaptic membrane against S4 (19S RP) at different time points of activation, indicating the fold of change respect 5 min of activation, and the graph associated show the measurement of the immunoblot. 2-way ANOVA with Sidak's *post-test* was performed for all statistical analysis. Mean with SEM bars are shown.

So far, we have shown that proteasome activity regulates B cell polarity by controlling the levels of actin at the centrosome and at the synaptic membrane. We then aimed to characterize the localization and activity of this complex in resting and activated B cells. For this, two different subunits of the constitutive 26S proteasome were labeled in B cells activated with antigen-coated beads for different time points: S4 for the regulatory subunit (19S RP) and α-β for the core particle (20S proteasome). Interestingly, central and cortical pools corresponding to proteasome subunits were observed (Figure 5A), where the central pool was located closely to the centrosome of B cells and upon activation progressively accumulated at the antigen-bead contact site (Figures 5B,C). At the synaptic interface, the proteasome was distributed toward the cell boundaries at earlier times of activation and later appeared at the center of the IS together with the centrosome (Figures S5A,B). Given the impact of proteasome inhibitors on actin dynamics, we labeled both F-actin and the 19S RP to study their co-distribution in resting and activated B cells with antigen-coated beads. Interestingly, whereas in resting B cells, significant pools of the proteasome and actin were found at the centrosome, both labels did not co-localize when recruited at the antigen contact site upon activation (Figure 5D). This was consistent with observations made at the synaptic membrane of B cells, where the proteasome was positioned adjacently to the boundaries of the F-actin peripheral ring, but no co-localization between both structures was observed (Figure S5A), suggesting that the proteasome could be locally restricting actin polymerization. We next assessed proteasome activity in resting and activated B cells and did not detect any major differences between both conditions (Figure S1A). However, proteasome activity measured in centrosome-rich fractions from B cells revealed that it decreased upon activation, without triggering major changes in its mass (Figures 5E–G). Accordingly, we found an increase in ubiquitinated proteins concentrated at centrosome of activated B cells, which could result from a lower proteasome activity at this level (Figure S6). Concomitantly to the depletion of the proteasome from the centrosome, we detected an accumulation of this complex in synaptic membranes isolated from activated B cells (Figure 5H), suggesting that the proteasome could be distributed from the polarized centrosomes to the synaptic membrane. Overall, our results reveal that the proteasome is recruited to the immune synapse of B cells upon activation and suggests that it could locally restrict sites of actin polymerization.

## Discussion

Our work reveals that proteasome activity regulates B cell polarity during immune synapse formation. Here, we show that B cells possess two pools of the 26S proteasome, which modulate actin dynamics both at the centrosome and at the IS. Proteasome activity is required for B cells to re-position their centrosome and thus facilitates antigen extraction by local lysosome secretion at the immune synapse.

The localization and activation of the proteasome has been shown to negatively regulate actin accumulation in neurons during axon cone growth (24). Thus, we initially expected that the inhibition of proteasome activity in B cells would lead to exacerbated spreading during their activation. However, our results show that proteasome inhibition impairs B cell spreading on antigen-coated surfaces, which can be explained by the lack of F-actin and its nucleator Arp2/3 at the immune synapse, observed under these conditions. Notably, B cells with inhibited proteasome also displayed higher amounts of actin and Arp2/3 at the centrosome, which are not efficiently depleted upon activation. Indeed, previous work has shown that the pools of actin at the centrosome and immune synapse are related, where recruitment of F-actin and Arp2/3 at the synaptic membrane is associated to their partial depletion from the centrosome (31). Intriguingly, we show that the proteasome is located at the centrosome of resting B cells, where ubiquitinated proteins are accumulated upon BCR stimulation. Thus, an appealing possibility is that the proteasome exerts its function by degrading ubiquitinated proteins involved in actin polymerization, thereby promoting actin depletion and centrosome re-positioning toward the immune synapse. Such a mechanism could allow B cells to rapidly establish a polarized phenotype in response to extracellular antigens.

A potential proteasome target could be the hematopoietic lineage cell-specific protein 1 (HS1), which recruits Arp2/3 and triggers actin polymerization during cell migration and chemotaxis (35, 36). When B cells are activated, HS1 is phosphorylated (pHS1) and relocated from the centrosome to the IS, but the mechanisms underlying this event are not yet well-understood. We propose that pHS1 could be specifically degraded by the proteasome at the centrosome, facilitating its accumulation at the IS, re-focusing actin polymerization from the centrosome to the IS. This idea is supported by the fact that HS1 has 5 ubiquitin sites (K34, K60, K123, K192, and K239) (37). Another protein that regulates Arp2/3 is the Wiskott–Aldrich syndrome protein (WASp), which is ubiquitinated by Cbl and degraded via proteasome, upon TCR stimulation. Defective WASp ubiquitination leads to altered actin dynamics, impaired cell spreading and calcium signaling in T cells, highlighting how degradation of proteins involved in actin polymerization regulate lymphocyte activation (38–40). Similarly to T cells, it is possible that the stabilization of WASp at the synapse, triggered by inhibition of proteasome activity, is responsible for the defective spreading observed in B cells. Accordingly, in B and T lymphocytes, deletion of WIP (Wasp-interacting protein), which protects WASp from degradation by the proteasome (38), leads to impaired cortical actin organization and defects in membrane protrusions upon activation (41).

We also found a higher accumulation of Syk and a more dispersed distribution of phospho-Syk at the IS of B cells with inhibited proteasome. Higher levels of Syk most likely result from impaired proteasome-dependent degradation of signaling molecules, which become rapidly ubiquitinated upon BCR stimulation (12, 34). Interestingly, although the amount of Syk was elevated upon proteasome inhibition, this was not accompanied by higher phosphorylated rates, suggesting a compensatory regulation of BCR signaling, possibly by phosphatases or autophagy (42–44). Moreover, upon proteasome inhibition, we found that pSyk became distributed beyond the peripheral actin ring, suggesting that proteasome activity at the synaptic membrane acts to confine signaling components to the center of the immune synapse. Similarly, in neurons, proteasome-mediated degradation is also used to remodel the post-synaptic density (PSD), where scaffold proteins are selectively degraded to regulate synaptic signaling. For instance, inhibition of proteasome activity or recruitment to the PSD triggers the accumulation of GluA2, a subunit of AMPAR, thereby enhancing synaptic transmission (17). Thus, synaptic proteostasis emerges as an important mechanism to regulate the signaling in specialized domains within different cell types (17, 45, 46).

The present findings reveal a new role for the proteasome in regulating the extraction and presentation of extracellular antigens by B lymphocytes. We show that the local distribution of proteasome balances the intracellular pools of actin, which has an impact on B cell polarity and organization of signaling components at the B cell synapse. These new findings contribute to the understanding of how drugs that target the proteasome can impact the activation of B lymphocytes in normal and pathological conditions and can be extrapolated to other cells of the immune system.

## Author Contributions

JI-V designed, performed, and analyzed most of the experiments, assembled figures, and participated in the writing of the manuscript. FD performed immunofluorescence and biochemical experiments and participated in the writing of the manuscript. JS performed immunofluorescence analysis and reviewed the manuscript. M-IY wrote the manuscript. M-IY and AS proposed the original hypothesis, designed experiments, supervised and funded the overall research.

### Conflict of Interest Statement

The authors declare that the research was conducted in the absence of any commercial or financial relationships that could be construed as a potential conflict of interest.

## Supplementary Material

Figure S1 Inhibition of proteasome activity in B cells by MG-132 and cell viability. **(A)** Proteasome activity measured in B cells pre-treated or not with MG-132 at resting and activated conditions. n.s. *N* = 4. **(B,D)** Immunoblot showing total levels of ubiquitinated proteins and actin in B cells (IIA1.6 cell line) treated with MG132 or Epoxomicin, respectively. **(C,E)** Cell viability of B cells treated with different concentrations of MG-132 or Epoxomicin for 2 h at 37°C, respectively. The number of trypan blue negative cells after incubation is shown. n.s. *N* = 4. **(F)** Representative images of control, MG-132 and Epoxomicin pre-treated primary B cells incubated with beads coated with anti-IgM+OVA (BCR-Ligand+) in resting (0 min) and activated (60 min) conditions. Fixed cell-bead conjugates were stained for OVA (green) and LAMP-1 (red). Scale bar = 10 μm. **(G)** Antigen extraction was measured as the amount of OVA extracted from the bead (see Materials and Methods). ^*^*p* < 0.05. *N* = 1 (>40 cells). 2-way ANOVA with Sidak's *post-test* or Student's *t*-test was performed for all statistical analysis. Mean with SEM bars are shown.

Figure S2 Re-positioning of the centrosome to the immune synapse is controlled by proteasome activity. Quantification of centrosome polarity indexes in B cells pre-treated or not with MG-132 and Epoxomicin under resting (0 min) or activated (60 min) conditions. *N* = 5. (100> cells). ^**^0.001 < *p* < 0.01, ^****^*p* < 0.001 2-way ANOVA with Sidak's *post-test*. Mean with SEM bars are shown.

Figure S3 Actin and Arp2 recruitment at the immune synapse depends on proteasome activity. **(A,B)** Representative images of control and MG-132 treated B cells activated with antigen-coated beads for different time points. Cells were stained for Arp2 and Phalloidin, respectively. Scale bar = 10 μm. **(C)** Representative images of control and MG-132 treated primary B cells activated on antigen-coated cover-slides for different time points. F-actin (green) and α-Tubulin (red). Scale bar = 10 μm. **(D)** Quantification of the spreading area of primary B cells pre-treated or not with MG-132 and activated for different time points. ^*^*P* < 0.05, ^****^*p* < 0.001. *N* = 2 (>100 cells). 2-way ANOVA with Sidak's *post-test* was performed.

Figure S4 Proteasome activity controls accumulation of Syk at the synaptic membrane. **(A)** B cell synaptic membranes analyzed by immunoblot for phosphorylated Syk (pSyk) and total Syk at different time points of activation for control and MG-132 treated B cells. **(B,C)** Quantification of Syk levels from immunoblots are shown and calculation of the pSyk/Syk ratio.

Figure S5 Localization of the proteasome at the synaptic membrane negatively correlates with actin accumulation at the immune synapse. **(A)** Confocal images of control and MG-132 treated B cells activated on antigen coated cover-slides for different time points. Labeling for Phalloidin (Green), 19S RP (Red) and α-Tubulin (Blue) is shown. White arrows indicate centrosome localization. Scale bar = 10 μm. **(B)** Quantification of 19S RP recruitment to the center of the immune synapse (see Materials and Methods). ^**^0.001 < *p* < 0.01, ^****^*p* < 0.001. *N* = 4. (>100 Cell). 2-way ANOVA with Sidak's *post-test*. Mean with SEM bars are shown.

Figure S6 B cell activation increases the accumulation of ubiquitinated proteins at the centrosome. Western blot showing total levels of ubiquitinated proteins in centrosome fractions isolated from resting and activated B cells pretreated or not with MG-132. Red rectangles indicate centrosome-rich fractions.

Video S1 Control B cell platted on antigen/B220 coated cover-slide expressing LifeAct-mCherry. Imaging acquisition was performed by TIRFM (see materials and methods).

Video S2 MG-132 treated B cell platted on antigen/B220 coated cover-slide expressing LifeAct-mCherry. Imaging acquisition was performed by TIRFM (see materials and methods).
