# Supplementary figures and images for: Proteasome Dependent Actin Remodeling Facilitates Antigen Extraction at the Immune Synapse of B Cells
Source: Front Immunol. 2019 Feb 19;10:225. doi: 10.3389/fimmu.2019.00225 (PMC6401660; doi:10.3389/fimmu.2019.00225)

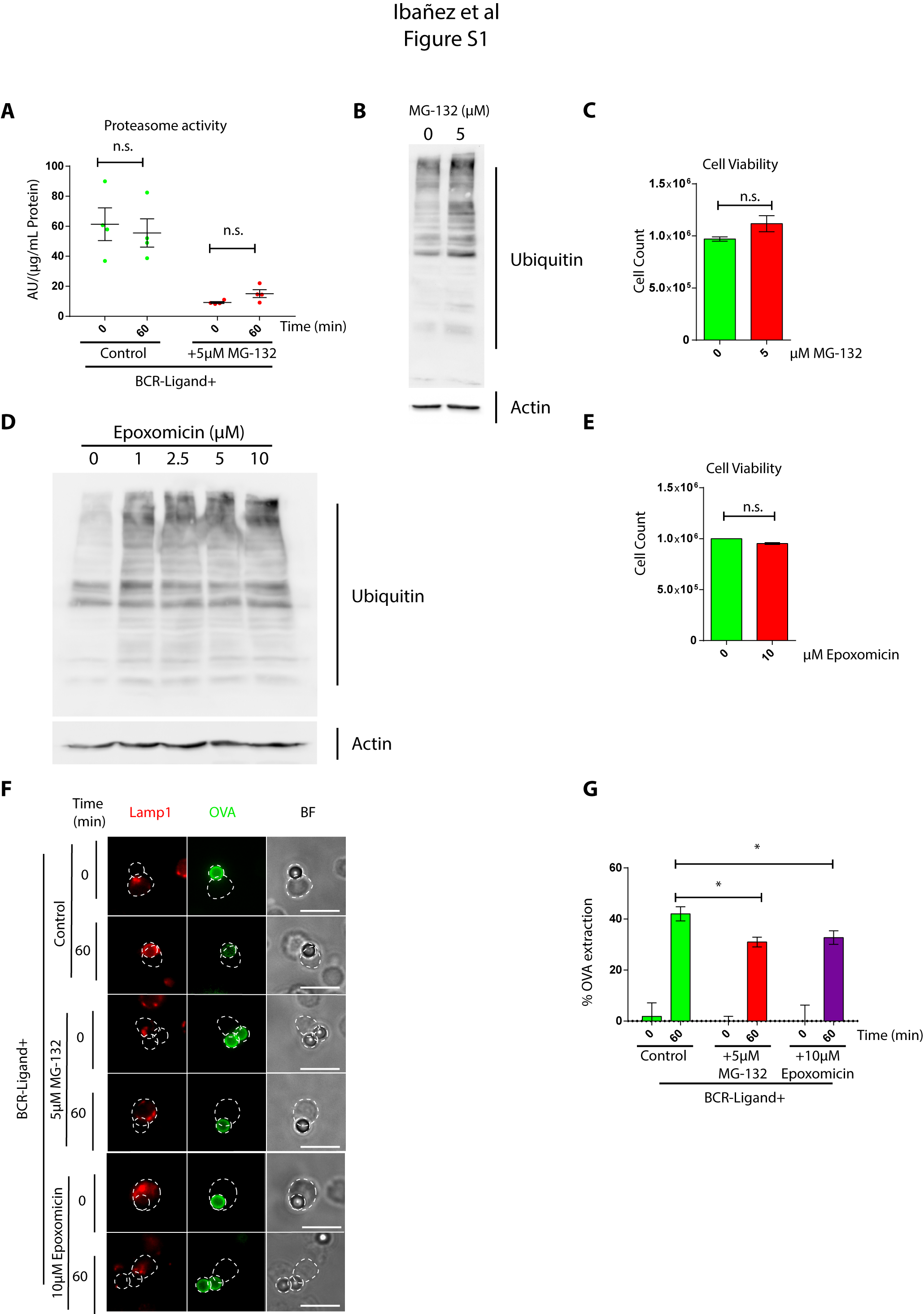

Supplement: Figure S1 — Inhibition of proteasome activity in B cells by MG-132 and cell viability. (A) Proteasome activity measured in B cells pre-treated or not with MG-132 at resting and activated conditions. n.s. N = 4. (B,D) Immunoblot showing total levels of ubiquitinated proteins and actin in B cells (IIA1.6 cell line) treated with MG132 or Epoxomicin, respectively. (C,E) Cell viability of B cells treated with different concentrations of MG-132 or Epoxomicin for 2 h at 37°C, respectively. The number of trypan blue negative cells after incubation is shown. n.s. N = 4. (F) Representative images of control, MG-132 and Epoxomicin pre-treated primary B cells incubated with beads coated with anti-IgM+OVA (BCR-Ligand+) in resting (0 min) and activated (60 min) conditions. Fixed cell-bead conjugates were stained for OVA (green) and LAMP-1 (red). Scale bar = 10 μm. (G) Antigen extraction was measured as the amount of OVA extracted from the bead (see Materials and Methods). *p < 0.05. N = 1 (>40 cells). 2-way ANOVA with Sidak's post-test or Student's t-test was performed for all statistical analysis. Mean with SEM bars are shown. [file Image_1.TIF]

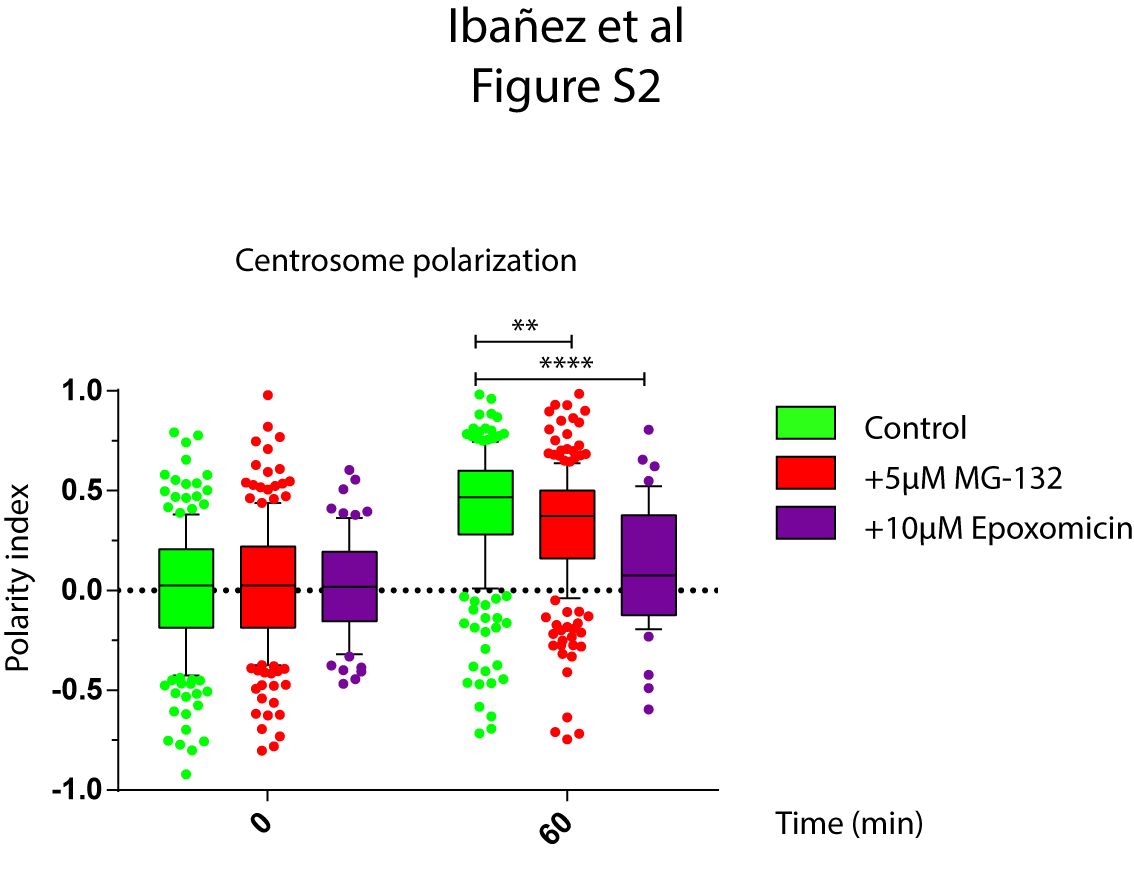

Supplement: Figure S2 — Re-positioning of the centrosome to the immune synapse is controlled by proteasome activity. Quantification of centrosome polarity indexes in B cells pre-treated or not with MG-132 and Epoxomicin under resting (0 min) or activated (60 min) conditions. N = 5. (100> cells). **0.001 < p < 0.01, ****p < 0.001 2-way ANOVA with Sidak's post-test. Mean with SEM bars are shown. [file Image_2.TIF]

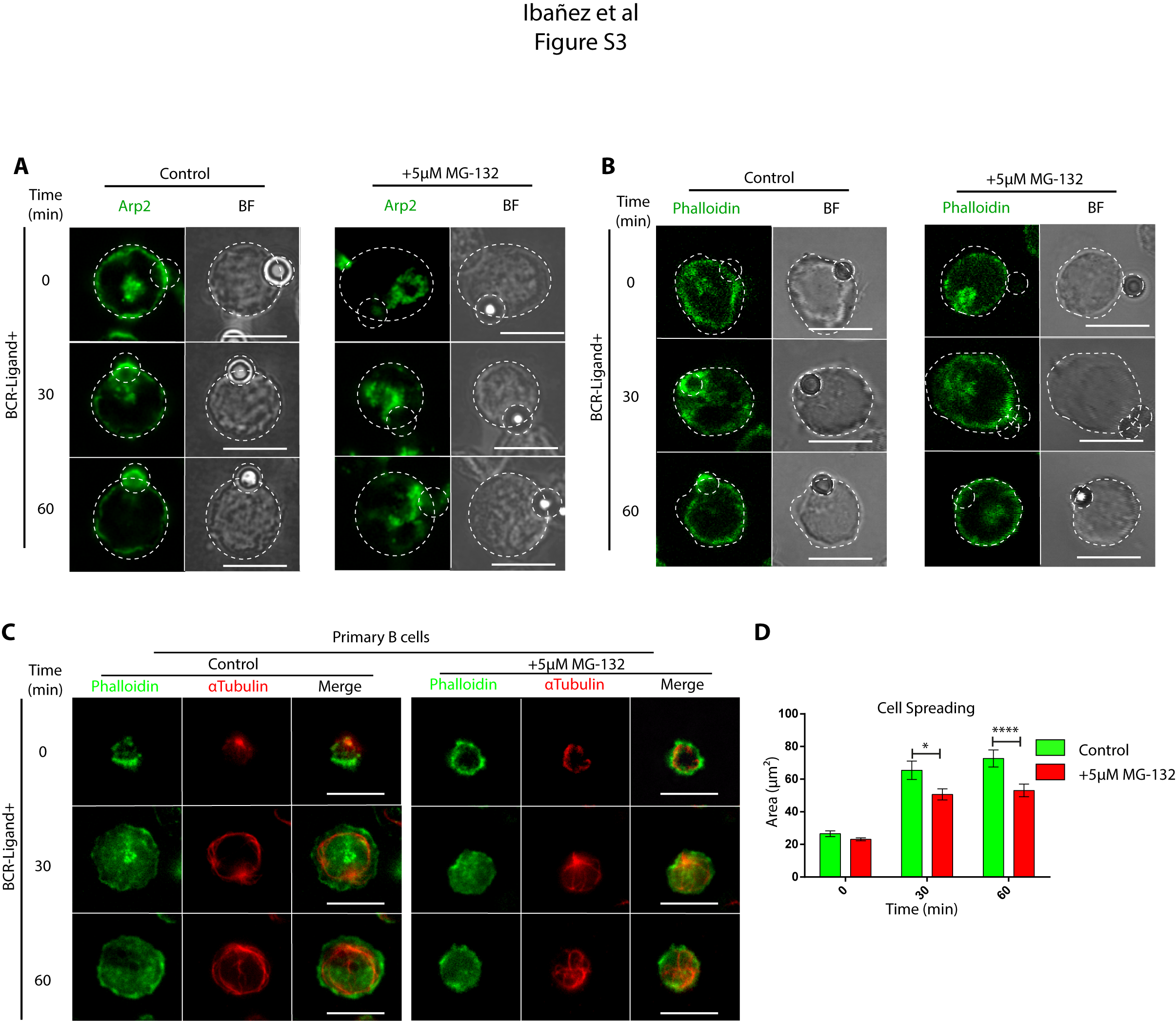

Supplement: Figure S3 — Actin and Arp2 recruitment at the immune synapse depends on proteasome activity. (A,B) Representative images of control and MG-132 treated B cells activated with antigen-coated beads for different time points. Cells were stained for Arp2 and Phalloidin, respectively. Scale bar = 10 μm. (C) Representative images of control and MG-132 treated primary B cells activated on antigen-coated cover-slides for different time points. F-actin (green) and α-Tubulin (red). Scale bar = 10 μm. (D) Quantification of the spreading area of primary B cells pre-treated or not with MG-132 and activated for different time points. *P < 0.05, ****p < 0.001. N = 2 (>100 cells). 2-way ANOVA with Sidak's post-test was performed. [file Image_3.TIF]

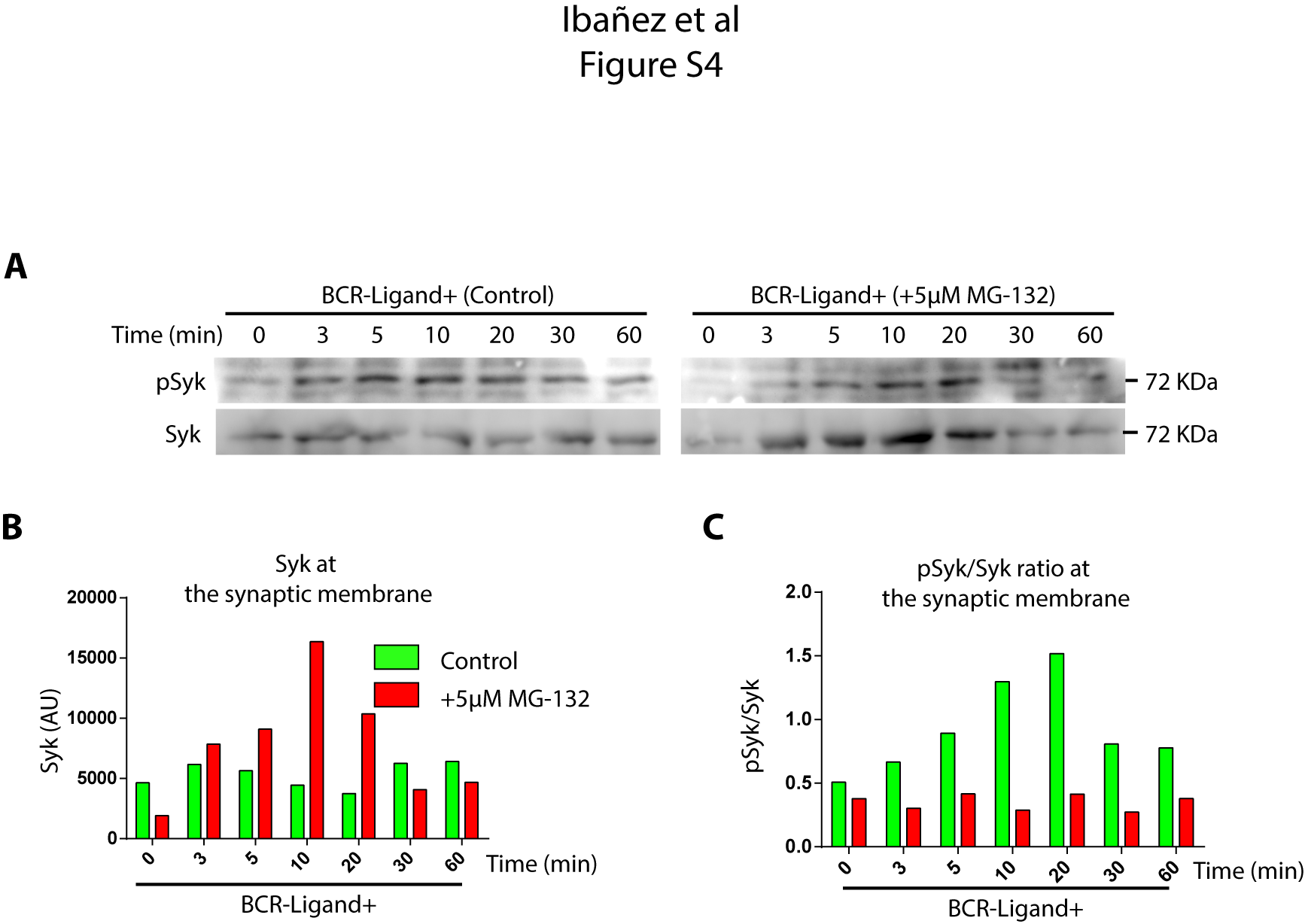

Supplement: Figure S4 — Proteasome activity controls accumulation of Syk at the synaptic membrane. (A) B cell synaptic membranes analyzed by immunoblot for phosphorylated Syk (pSyk) and total Syk at different time points of activation for control and MG-132 treated B cells. (B,C) Quantification of Syk levels from immunoblots are shown and calculation of the pSyk/Syk ratio. [file Image_4.TIF]

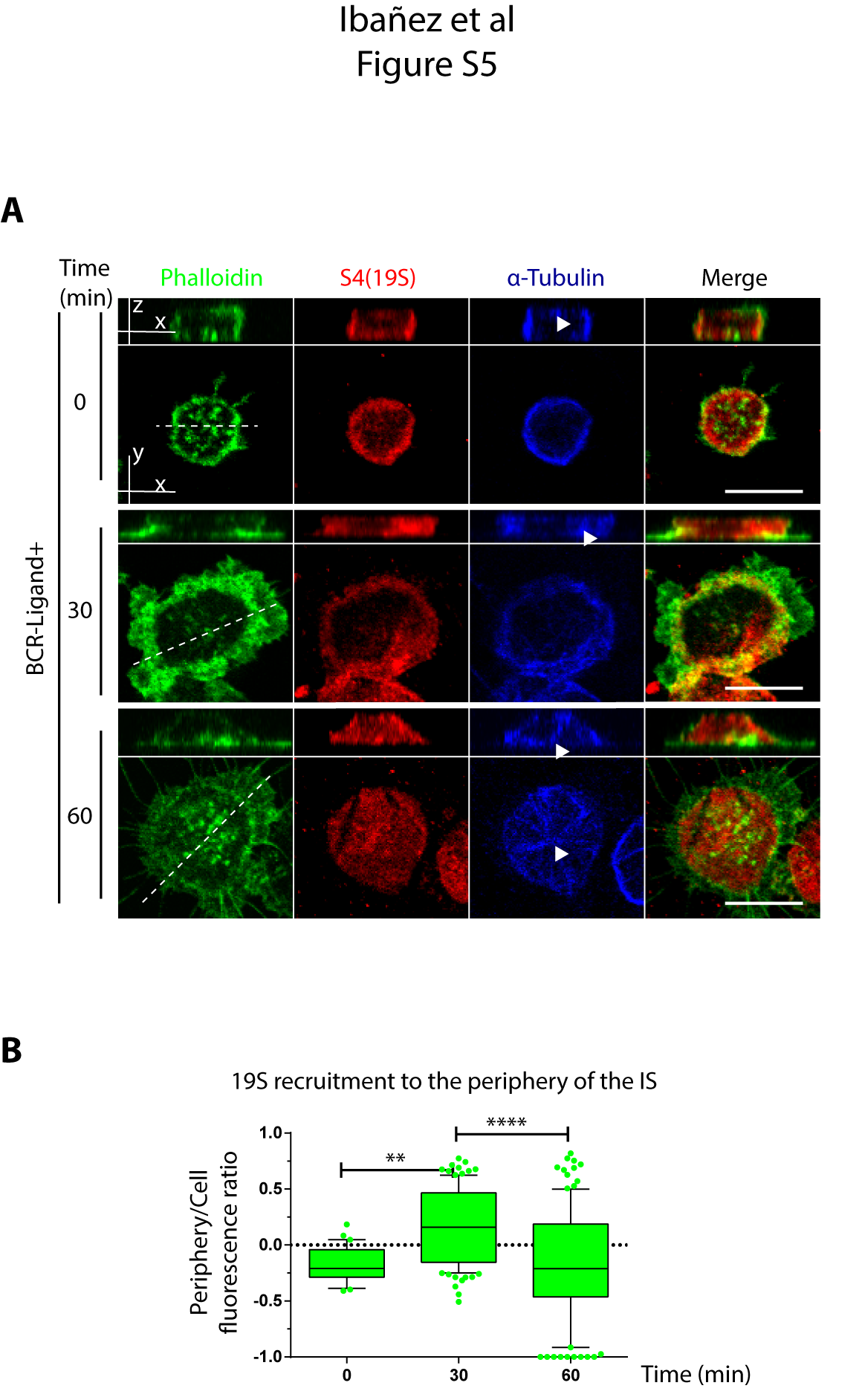

Supplement: Figure S5 — Localization of the proteasome at the synaptic membrane negatively correlates with actin accumulation at the immune synapse. (A) Confocal images of control and MG-132 treated B cells activated on antigen coated cover-slides for different time points. Labeling for Phalloidin (Green), 19S RP (Red) and α-Tubulin (Blue) is shown. White arrows indicate centrosome localization. Scale bar = 10 μm. (B) Quantification of 19S RP recruitment to the center of the immune synapse (see Materials and Methods). **0.001 < p < 0.01, ****p < 0.001. N = 4. (>100 Cell). 2-way ANOVA with Sidak's post-test. Mean with SEM bars are shown. [file Image_5.TIF]

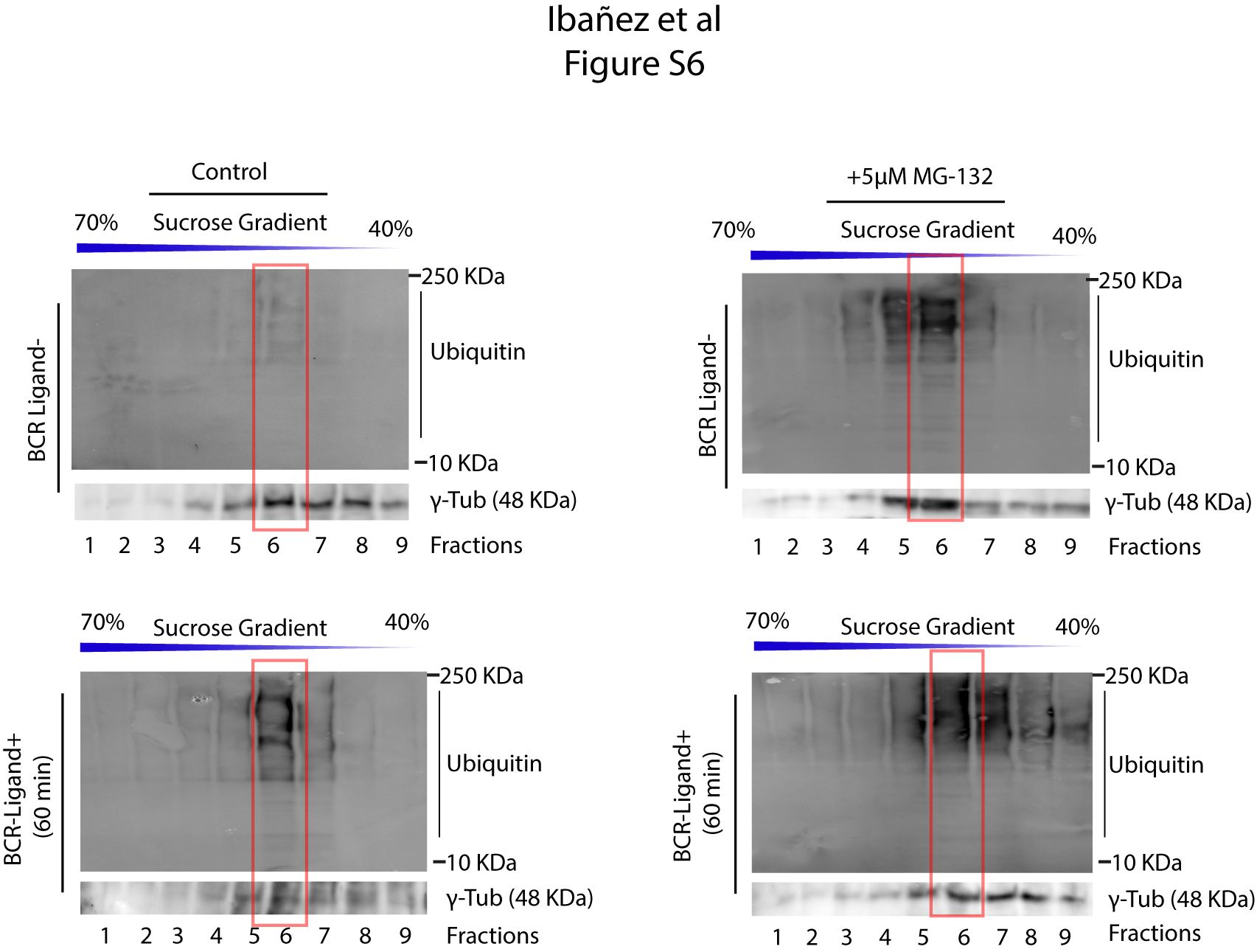

Supplement: Figure S6 — B cell activation increases the accumulation of ubiquitinated proteins at the centrosome. Western blot showing total levels of ubiquitinated proteins in centrosome fractions isolated from resting and activated B cells pretreated or not with MG-132. Red rectangles indicate centrosome-rich fractions. [file Image_6.TIF]
